# Low-Intensity Pulsed Ultrasound Enhances Intramembranous Bone Healing in a Critical-Size Bone Defect of the Rat Calvaria

**DOI:** 10.3390/jcm15124595

**Published:** 2026-06-13

**Authors:** Darian Volarić, Gordana Žauhar, Jie Chen, Ana Terezija Jerbić Radetić, Rok Kostanjšek, Hrvoje Omrčen, Silvije Šegulja, Edi Rođak, Olga Cvijanović Peloza

**Affiliations:** 1 Department of Physical Medicine and Rehabilitation, Thalassotherapia Crikvenica—Special Hospital for Medical Rehabilitation, Gajevo Šetalište 21, 51260 Crikvenica, Croatia; 2 Department of Medical Physics and Biophysics, Faculty of Medicine, University of Rijeka, Braće Branchetta 20/1, 51000 Rijeka, Croatia; gordana.zauhar@uniri.hr; 3 Faculty of Physics, University of Rijeka, Radmile Matejčić 2, 51000 Rijeka, Croatia; 4 College of Biomedical Engineering, Fudan University, Shanghai 200433, China; jc65@ualberta.ca; 5 Department of Electrical and Computer Engineering, University of Alberta, Edmonton, AB T6G 2V4, Canada; 6 Department of Anatomy, Faculty of Medicine, University of Rijeka, Braće Branchetta 20/1, 51000 Rijeka, Croatia; ana.jerbic.radetic@uniri.hr (A.T.J.R.); olga.cvijanovic@medri.uniri.hr (O.C.P.); 7 Department of Biology, Biotechnical Faculty, University of Ljubljana, Jamnikarjeva 101, 1000 Ljubljana, Slovenia; rok.kostanjsek@bf.uni-lj.si; 8 Department of Clinical Microbiology, Teaching Institute of Public Health of Primorsko-Goranska County, Krešimirova 52a, 51000 Rijeka, Croatia; hrvoje.omrcen@zzjzpgz.hr; 9 Department of Clinical Sciences I, Faculty of Health Studies, University of Rijeka, Viktora Cara Emina 5, 51000 Rijeka, Croatia; silvije.segulja@fzsri.uniri.hr; 10 Department of Histology and Embryology, Faculty of Medicine Osijek, University of Osijek, J. Huttlera 4, 31000 Osijek, Croatia; erodak@mefos.hr

**Keywords:** low-intensity pulsed ultrasound, autologous bone graft, critical-size bone defect, rat calvarial defect, bone healing, intramembranous ossification, micro-computed tomography, TNF-α, osteogenesis

## Abstract

**Objectives**: Low-intensity pulsed ultrasound (LIPUS) has been widely utilized as a biophysical modality accelerating fracture healing, particularly in bones undergoing endochondral ossification. However, its efficacy in facilitating intramembranous ossification remains unclear. This study aimed to evaluate the effects of LIPUS and autologous bone (AB) on bone healing in a critical-size bone defect (CSBD) model of the rat calvaria. **Methods**: We performed micro-computed tomography (micro-CT) and immunohistochemical TNF-α analysis on bone specimens to assess osteogenesis. **Results**: Micro-CT demonstrated significant increases in newly formed bone on day 30 compared with days 7 and 15 across all groups (*p* < 0.001). The highest bone volume was observed in the AB group (26.83%), followed by the LIPUS group (23.74%), and the lowest in the control (15.85%). Immunohistochemical analysis revealed significantly higher TNF-α expression on day 7 in the control group (172.0 ± 1.1) than in the AB (133.8 ± 0.9) and LIPUS (125.2 ± 0.8) groups (*p* < 0.001). On day 15, TNF-α expression was significantly higher in the LIPUS group (137.7 ± 1.3) than in both the AB (134.2 ± 1.8) and control (126.6 ± 2.2) groups (*p* < 0.001). At day 30, TNF-α levels in the LIPUS group (147.6 ± 1.9) remained significantly higher than in the control group (115.8 ± 0.9) (*p* < 0.001), with no significant difference compared to the AB group (146.3 ± 0.8). **Conclusions**: Although AB grafting achieved the greatest bone volume, LIPUS demonstrated considerable regenerative potential and may represent a promising non-invasive therapeutic approach to enhance intramembranous bone regeneration.

## 1. Introduction

Fracture repair represents a multifaceted biological process. From a clinical perspective, successful healing depends on precise realignment of the displaced bone fragments and effective mechanical stabilization. During bone healing, the repair process progresses through a series of well-defined stages. Initially, a blood clot forms at the site of injury, followed by an inflammatory phase, the development of granulation tissue, callus formation, and ultimately the remodeling phase [1]. It is important to note that both endochondral and intramembranous ossification contribute to post-injury bone regeneration; however, only the hard callus is formed directly through the deposition and mineralization of type I collagen secreted by osteoblasts without a preceding cartilage template [2].

At the fracture site, a blood clot forms through plasma coagulation and platelet activation. This provisional matrix recruits neutrophils, which secrete proinflammatory chemokines, such as interleukin-6 (IL-6), thereby attracting monocytes and macrophages. Macrophages subsequently remove the temporary fibrin matrix and necrotic cells via phagocytosis. In addition to clearing cellular and extracellular debris, they release inflammatory and chemotactic mediators, including tumor necrosis factor alpha (TNF-α), IL-6, and interleukin-1 (IL-1). During this inflammatory stage, cyclooxygenase-2 (COX-2) expression is upregulated, promoting prostaglandin synthesis and facilitating the recruitment and migration of inflammatory cells to the fracture site.

Chemotactic cytokines further recruit mesenchymal stem cells (MSCs) and fibroblasts, which contribute to tissue repair after the resolution of the acute inflammatory response. MSCs secrete various bone morphogenetic proteins (BMPs) that regulate proliferation, mitogenesis, angiogenesis, and chemotaxis. Following the inflammatory phase, the local hypoxic environment stimulates the formation of granulation tissue, which is rich in progenitor cells and newly formed vasculature, arising from a disorganized extracellular matrix [3]. During the late inflammatory stage, a soft callus forms, comprising cartilaginous and connective tissue matrices, immature bone, chondrocytes, and fibroblasts derived from progenitor cells. The soft callus provides initial mechanical support, after which the cartilage is resorbed and chondrocytes undergo apoptosis. Callus formation is associated with a progressive decline in proinflammatory cytokines and a concomitant increase in Runx2 and Osterix (Osx) expression, two key transcriptional regulators of osteoblast differentiation [4,5].

Gradually, the extracellular matrix mineralizes, replacing the cartilaginous callus with a hard callus. Around two weeks after fracture, neoangiogenesis reaches its peak, coinciding with elevated vascular endothelial growth factor (VEGF) expression. Osteogenesis is most pronounced between days 14 and 21, during which a second rise in inflammatory cytokines (TNF-α, IL-6, IL-1) occurs, corresponding to bone turnover and remodeling. From day 21 to 35, immature bone continues to mature into mineralized, structurally organized bone [6]. Over time, the hard callus develops a lamellar architecture characteristic of mature bone, marking the remodeling phase [2].

Various biophysical modalities have been investigated as adjunctive therapies to enhance compromised bone healing. Among these, pulsed electromagnetic fields (PEMFs), combined magnetic fields, direct electrical stimulation, and low-intensity pulsed ultrasound (LIPUS) have received considerable attention.

Pulsed electromagnetic systems deliver asymmetric, quasi-rectangular pulses in bursts, with individual pulse durations of approximately 260 μs, burst frequencies of 15 Hz, and peak magnetic field strengths of 20 mT. Clinical application of these systems for an average of 7.1 h per day has been associated with nonunion healing rates of approximately 80%, while alternative PEMF protocols involving treatment durations of up to 10 h daily achieved success rates of 63.5% [7].

Devices generating combined static and dynamic magnetic fields produce a constant 20 μT magnetic field superimposed with a sinusoidal dynamic field at 76.6 Hz and ±40 μT amplitude. Daily exposure of 30 min yielded a nonunion healing rate of 60.7% [8].

Implanted direct current stimulators deliver a continuous current of 20 μA via a cathodal wire for 24 h per day over a minimum of six months, achieving long-term nonunion repair rates ranging from 38.8% to 66.7% [9].

Low-intensity pulsed ultrasound (LIPUS) is a specialized ultrasound modality characterized by the delivery of acoustic energy at low power levels in an intermittent (pulsed) manner. Its spatial average temporal average intensity (I_SATA_) generally remains below 100 mW/cm^2^. Contemporary clinical devices most commonly operate at an intensity of 30 mW/cm^2^ (SATA), with a frequency of 1.5 MHz, a pulse repetition rate of 1 kHz, and a 20% duty cycle. At such low energy output, cavitational effects are not expected to occur, and the associated temperature rise is considered biologically negligible [10].

Ultrasound waves are mechanical longitudinal waves which are generated by cyclical pressure fluctuations in a medium at frequencies exceeding the upper threshold of human hearing, typically above 20 kHz. In medicine, acoustic energy within this frequency range is applied across a broad spectrum of applications, with its biological effects largely determined by output intensity and delivery parameters. Low-power emissions are widely employed for diagnostic imaging, moderate intensities are utilized in rehabilitative and physiotherapeutic interventions, whereas high-intensity focused ultrasound is capable of inducing precise thermal ablation for surgical purposes [11].

Although diagnostic ultrasound, conventional therapeutic ultrasound, and LIPUS may operate within comparable intensity ranges, their interactions with biological tissues are not equivalent. Variations in waveform modulation, temporal patterning, and energy distribution result in distinct cellular and molecular responses, underscoring the functional differences between these ultrasound modalities [12].

The concept of enhancing bone repair through LIPUS was first introduced by Xavier and Duarte in 1983 [13]. Subsequent clinical investigations demonstrated accelerated healing in acute fractures treated with LIPUS. These findings ultimately led to regulatory approval in the United States, where the Food and Drug Administration (FDA) authorized commercial LIPUS devices for the treatment of fresh fractures in 1994 and for established nonunions in 2000 [14]. Beyond skeletal applications, LIPUS has also shown regenerative potential in soft tissues, including tendons, ligaments, and cartilage, primarily through the stimulation of fibroblast activity, collagen synthesis, and enhanced angiogenic, chondrogenic, and osteogenic responses [15].

Despite extensive in vivo and in vitro evidence supporting the pro-regenerative effects of LIPUS on bone repair, the underlying biological mechanisms remain incompletely elucidated. Proposed explanations involve biomechanical interactions at the cellular and molecular levels, including acoustic radiation forces, microstreaming, surface wave propagation, fluid flow-mediated redistribution of nutrients, oxygen, and signaling molecules within the extracellular environment [12,16]. Mechanical stimulation induced by LIPUS has been shown to promote the commitment of mesenchymal stem cells (MSCs) to the osteogenic lineage and to enhance osteoid production and subsequent mineralization by bone-forming cells [17].

A critical-size bone defect (CSBD) is defined as the minimal osseous injury that fails to undergo complete spontaneous repair over the lifetime of the experimental animal [18]. In the context of rat calvarial models, defects measuring approximately 8 mm in diameter are widely recognized as critical-size defects. Because calvarial bone formation during healing recapitulates the intramembranous ossification observed in embryogenesis, defects in the rat calvaria provide a robust and physiologically relevant model for investigating intramembranous bone regeneration [19,20]. Although LIPUS has been extensively investigated in both experimental and clinical fracture healing, most available evidence originates from conditions in which endochondral ossification represents a major component of the regenerative process. Consequently, substantially less is known about the effects of LIPUS on intramembranous bone regeneration, particularly in critical-size bone defects that do not undergo complete spontaneous healing. Therefore, the rat calvarial CSBD model provides a valuable platform for investigating the regenerative potential of LIPUS in a predominantly intramembranous healing environment.

Autologous bone (AB) grafts remain the benchmark for promoting bone repair due to their combined osteoconductive, osteoinductive, and osteogenic capacities. Osteoconduction is facilitated by the graft’s three-dimensional matrix, which serves as a scaffold supporting the attachment and migration of osteoblasts and osteocytes, thereby guiding new bone formation from the defect center outward and influencing the rate of osteointegration. Osteoinduction arises from the presence of growth factors, extracellular matrix proteins, and signaling molecules within the graft, which collectively stimulate cellular pathways involved in bone regeneration. The osteogenic potential of AB is reflected in its ability to recruit and activate progenitor cells and mature osteoblasts, directly contributing to new bone synthesis. A notable limitation of AB grafts, however, is their rapid resorption; up to approximately 40% of the grafted bone volume can be lost during healing and remodeling. To mitigate this, AB grafts are frequently combined with xenografts, which provide structural support and resistance to resorption, thereby enhancing the stability of bone volume during regeneration [21].

Based on the previously reported regenerative effects of LIPUS and the established osteogenic properties of autologous bone grafts, we hypothesized that both interventions would enhance bone regeneration in rat calvarial CSBDs compared with spontaneous healing. Furthermore, we expected that the observed healing dynamics would be accompanied by differences in TNF-α expression among the experimental groups.

Given the limited understanding of CSBD healing dynamics following LIPUS treatment and the absence of direct comparisons with AB grafting, the primary objective of this study was to evaluate and compare the effects of LIPUS and AB on the repair of rat calvarial CSBD relative to spontaneous healing in the control group. The specific aims were as follows: (1) to establish a reproducible rat model of CSBD and assess healing at defined time points (days 7, 15, and 30); (2) to quantify three-dimensional bone parameters in harvested calvarial tissue using micro-computed tomography (micro-CT); and (3) to characterize the temporal expression of TNF-α within bone tissue through immunohistochemical analysis.

## 2. Materials and Methods

### 2.1. Materials

A total of 45 male Wistar rats were included in this experimental study. The animals were bred and maintained at the Department of Physiology, Pathophysiology and Immunology, Faculty of Medicine, University of Rijeka, Croatia. The animals were randomly allocated into three experimental groups (*n* = 15 per group). Within each group, five animals (*n* = 5) were euthanized and analyzed at each postoperative time point (days 7, 15, and 30). A CSBD was surgically created in all animals and subsequently covered with a collagen membrane serving as a periosteal substitute. The animals in the first group received LIPUS treatment only, those in the second group were treated with an autologous bone (AB) graft, whereas the third group was used as a control to evaluate spontaneous defect healing. The experimental protocol received approval from the Animal Welfare Committee, the Ethics Committee for Biomedical Research of the Faculty of Medicine, University of Rijeka, and the Ministry of Agriculture (EP 302/2021). All animal procedures were performed in compliance with national regulations governing the use of animals for scientific purposes.

**Figure 1:**
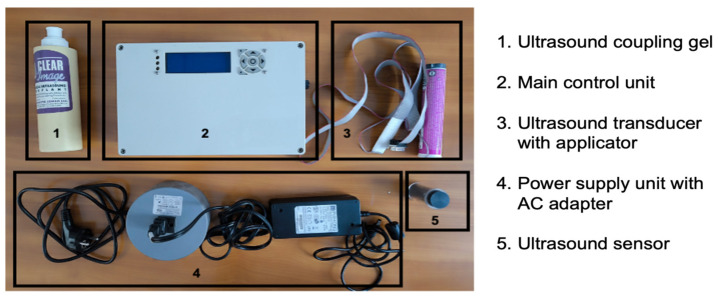
Components of the low-intensity pulsed ultrasound (LIPUS) device utilized for experimental treatment.

LIPUS waves were generated using the SonaCell device (IntelligentNano Inc., Edmonton, AB, Canada) (Figure 1). Treatment was performed with an ultrasound frequency of 1.5 MHz, a pulse repetition frequency of 1 kHz, a duty cycle of 20%, and a spatial average temporal average intensity of 30 mW/cm^2^. Insonation was applied using a circular transducer measuring 24 mm in diameter. To minimize movement during ultrasound application, the experimental animal was positioned in a custom-made wooden holder that stabilized the head and reduced excessive motion. Ultrasound gel was applied to the site of the calvarial defect, after which the LIPUS transducer was placed over the defect area. The transducer was maintained in a stable position throughout the insonation period to ensure consistent acoustic coupling and energy delivery. Ultrasound treatment was administered exclusively in the LIPUS experimental group according to a protocol of three sessions per week, with each session lasting 20 min. Treatment was initiated on the first postoperative day following CSBD creation and was continued until day 30, depending on the designated euthanasia time points. To minimize variability, all animals were treated using the same device settings, coupling method, transducer positioning, treatment duration, and application schedule.

An 8 mm CSBD was created in the frontoparietal region using a trephine bur operated at 1500 rpm. A comprehensive account of the surgical technique used has been previously reported [22].

In the AB group, the autologous bone graft was obtained from the calvarial bone, removed during creation of the 8-mm critical-size bone defect. The excised bone was mechanically particulated using a bone crusher (Knochenquetsche 67-680-000, Ustomed instruments, Tuttlingen, Germany) and immediately reimplanted into the defect site. The particulate graft was positioned within the defect and subsequently covered with a collagen membrane.

Following animal sacrifice, calvarial bone samples were excised and fixed in 4% paraformaldehyde at 4 °C. After fixation, the specimens were stored in 70% ethanol and transported for subsequent analyses.

### 2.2. Methods

#### 2.2.1. Micro-Computed Tomography (Micro-CT)

Calvarial specimens were examined using a micro-computed tomography scanner NeoScan N80 (NeoScan, Mechelen, Belgium). Image acquisition was performed at 58 kV and 200 µA with a voxel size of 10 µm, a rotation increment of 0.30°, and frame averaging of 2. A total of 659 projection images were obtained for each specimen. Reconstruction of the image datasets was carried out using NRecon software (version 2.0; Bruker, Kontich, Belgium), followed by morphometric analysis with CTAn software (version 2.0; Bruker, Kontich, Belgium). The assessed microarchitectural parameters included bone volume (BV/TV; %), trabecular thickness (Tb.Th; mm), and trabecular number (Tb.N; mm^−1^). For the analysis, an 8 mm diameter circular region of interest (ROI) corresponding to the original defect area was delineated to ensure standardized evaluation of bone regeneration. Quantitative image analysis was performed within the selected ROI. Newly formed mineralized tissue was identified and segmented using a grayscale threshold range of 50–255, which was applied consistently across all specimens. The segmented region was subsequently used for the calculation of BV/TV, Tb.Th, and Tb.N parameters.

#### 2.2.2. Immunohistochemical Analysis

Following micro-CT evaluation, calvarial specimens were decalcified in Osteofast 2 solution (Biognost, Zagreb, Croatia) for 48 h and subsequently processed for paraffin embedding. Serial sections measuring 3–5 μm in thickness were prepared using a rotary microtome (Leica RM 2155, Leica Instruments, Ballerup, Denmark).

Prior to immunostaining, paraffin sections were deparaffinized with xylene and rehydrated through descending ethanol concentrations (100%, 96%, and 75%). Endogenous peroxidase activity was blocked with 0.3% hydrogen peroxide diluted in methanol. Antigen retrieval was carried out in citrate buffer at 60 °C for 10 min. The sections were then incubated overnight with a primary anti-TNF-α antibody (ab270264, Abcam, Cambridge, UK; dilution 1:200). After rinsing, the samples were exposed to biotinylated secondary antibodies for 45 min at room temperature. Antigen localization was visualized using a streptavidin–peroxidase detection system (LSAB+ Kit, DakoCytomation, Glostrup, Denmark) with 3,3′-diaminobenzidine (DAB, DakoCytomation, Glostrup, Denmark) as the chromogenic substrate. Hematoxylin was used for nuclear counterstaining. Subsequently, the sections were dehydrated through increasing ethanol concentrations, cleared in xylene, and mounted with Entellan medium. Microscopic evaluation was performed using an Olympus BHA light microscope equipped with a 20× objective lens (Olympus Corporation, Tokyo, Japan) and a digital camera (Sony Corporation, Tokyo, Japan). Quantitative assessment of TNF-α immunostaining intensity was performed on the acquired images using ImageJ software (version 1.53; National Institutes of Health, Bethesda, MD, USA).

#### 2.2.3. Statistical Analysis

All statistical analyses were conducted using Statistica software version 14.0.1.25 (TIBCO Software Inc., Palo Alto, CA, USA). The normality of data distribution was assessed with the Kolmogorov–Smirnov test and confirmed for all evaluated variables. Consequently, the results are reported as mean values ± standard deviation (SD). Comparisons between experimental groups and time points were performed using one-way and two-way analysis of variance (ANOVA), as appropriate. When significant overall differences were identified, pairwise comparisons were carried out using the Scheffé post-hoc test. Homogeneity of variances was verified before conducting the analyses. Statistical significance was defined as *p* < 0.05.

## 3. Results

### 3.1. Micro-CT

Micro-CT scanning provided quantitative values of three-dimensional bone microarchitectural parameters, including bone volume (BV/TV; %), trabecular thickness (Tb.Th; mm), and trabecular number (Tb.N; mm^−1^).

The results demonstrated statistically significant differences among the experimental groups for all three parameters—BV/TV and Tb.Th (*p* < 0.001), as well as Tb.N (*p* < 0.05).

Micro-CT–derived BV/TV values at the respective time points are presented in Table 1. Analysis of variance (ANOVA) revealed statistically significant differences among all experimental groups at each evaluated time point (days 7, 15, and 30), with significance confirmed for every time point (*p* < 0.001). Subsequent post-hoc analysis using the Scheffé test demonstrated significant differences between all pairwise group comparisons at each individual time point (*p* < 0.001).

**Figure 2:**
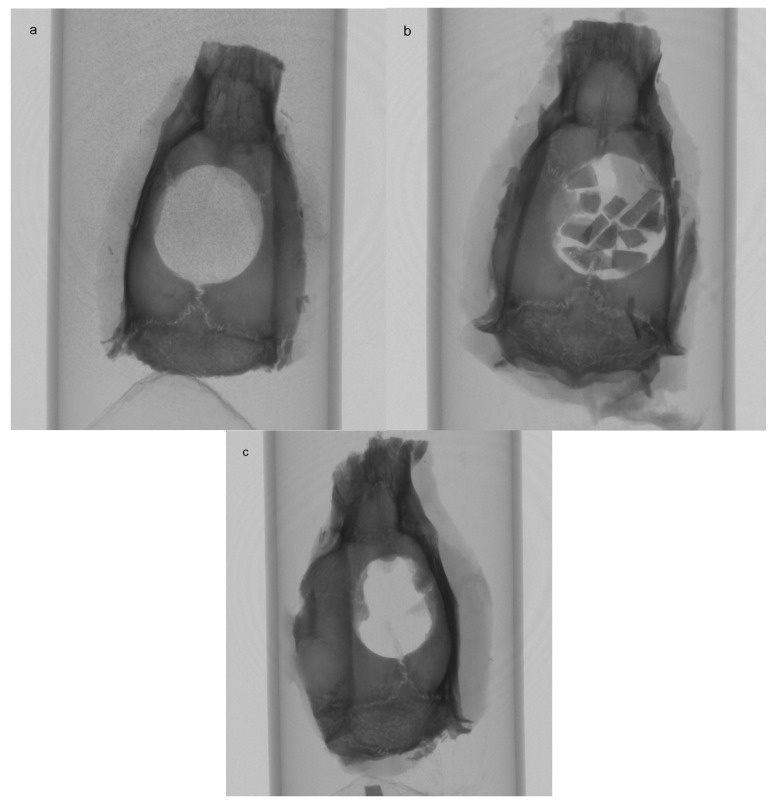
Representative micro-CT images obtained on day 30 of healing of the rat frontoparieto-occipital calvarial complex from the control group (**a**), autologous bone group (**b**), and LIPUS group (**c**).

Figure 2 presents representative micro-CT images of the frontoparieto-occipital calvarial complex in rats across all experimental groups.

**Figure 3:**
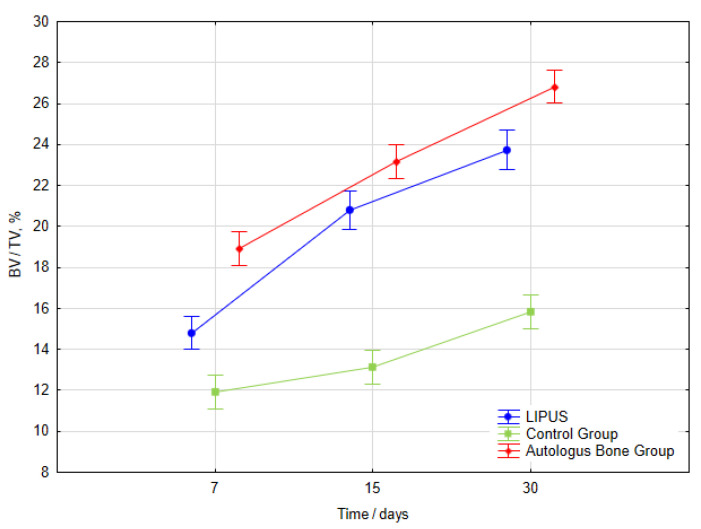
Temporal changes of the micro-CT parameter BV/TV in the LIPUS-treated group, autologous bone group, and control group.

Figure 3 illustrates the temporal progression of bone volume (BV/TV) across the evaluated time points (days 7, 15, and 30) in all groups. Statistical analysis demonstrated significant intragroup differences over time (*p* < 0.001), indicating a significant effect of time on BV/TV increase. The greatest temporal increase in BV/TV was observed in the AB group, followed by the LIPUS group, while the control group exhibited the smallest increase.

Micro-CT-derived Tb.Th values at the respective time points are presented in Table 2. Analysis of variance (ANOVA) revealed statistically significant differences among the experimental groups at all evaluated time points (days 7, 15, and 30) (*p* < 0.001). Subsequent post-hoc analysis using the Scheffé test demonstrated significant pairwise differences between the AB group and both the LIPUS and control groups at all time points, whereas a significant difference between the LIPUS and control groups was observed only on days 15 and 30.

**Figure 4:**
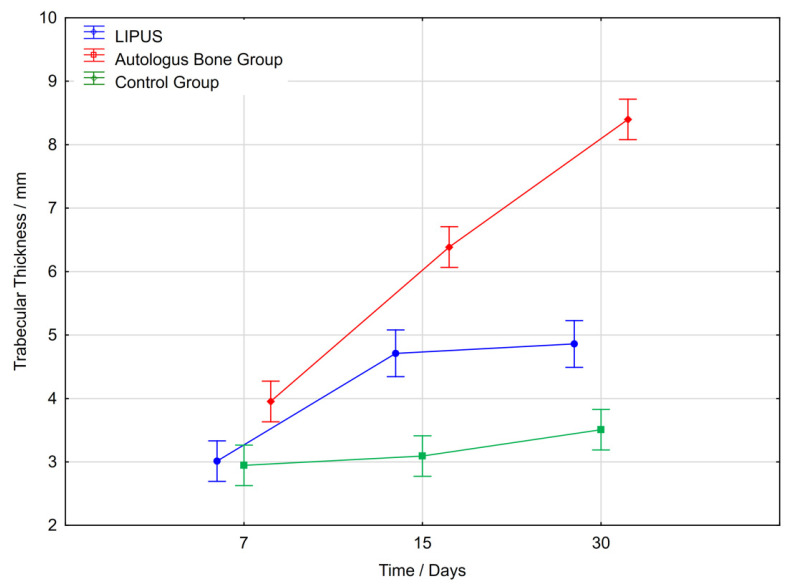
Temporal changes of the micro-CT parameter trabecular thickness in the LIPUS-treated group, autologous bone group, and control group.

Figure 4 depicts the temporal trend of trabecular thickness (Tb.Th) at days 7, 15, and 30 for all experimental groups. Significant intragroup differences were detected over time (*p* < 0.001), confirming a positive effect of time on the increase in trabecular thickness.

Micro-CT-derived Tb.N values at the respective time points are presented in Table 3. Analysis of variance (ANOVA) revealed statistically significant differences among the experimental groups at all evaluated time points (days 7, 15, and 30) (*p* ≤ 0.010). Subsequent post-hoc analysis using the Scheffé test demonstrated significant pairwise differences between all groups on days 7 and 15, whereas on day 30 a significant difference was observed only between the AB and LIPUS groups.

**Figure 5:**
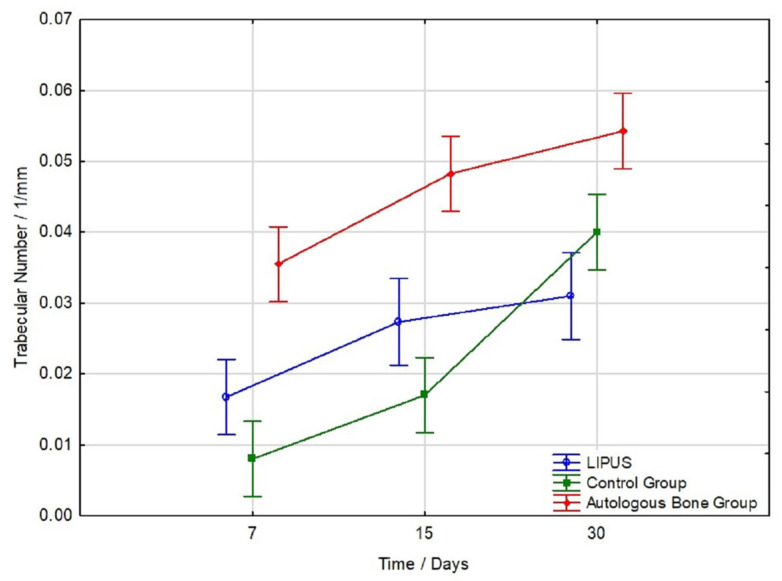
Temporal changes of the micro-CT parameter trabecular number in the LIPUS-treated group, autologous bone group, and control group.

Figure 5 shows the progression of trabecular number (Tb.N) across the same time points. Statistical analysis revealed significant time-dependent differences within each group (*p* < 0.001), demonstrating a positive influence of time on trabecular number.

### 3.2. Immunohistochemical Evaluation of TNF-α

The intensity scores obtained from TNF-α immunohistochemical staining are summarized in Table 4. Significant differences in TNF-α expression were identified among the experimental groups at all evaluated time points (days 7, 15, and 30; *p* < 0.001). Post-hoc comparisons demonstrated significant differences between all pairwise group combinations on days 7 and 15 (*p* < 0.001). At day 30, both the LIPUS and AB groups exhibited significantly higher TNF-α expression than the control group (*p* < 0.001), whereas no significant difference was detected between the two experimental groups.

**Figure 6:**
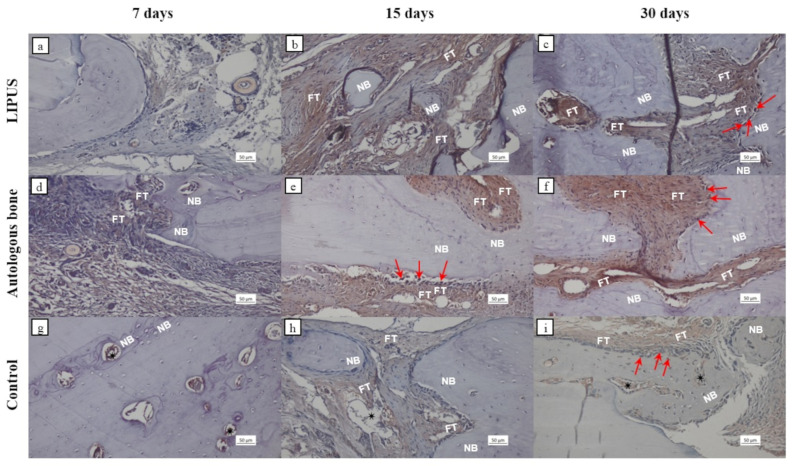
Representative TNF-α immunohistochemical staining in coronal sections of rat calvarial defects from the LIPUS (**a**–**c**), autologous bone (AB; **d**–**f**), and control (**g**–**i**) groups. Images were obtained at postoperative days 7 (**a**,**d**,**g**), 15 (**b**,**e**,**h**), and 30 (**c**,**f**,**i**) to demonstrate temporal changes in TNF-α expression during healing. The following structures are indicated: newly formed bone (NB), autologous bone (AB), fibrous tissue (FT), osteoblasts (red arrows), and blood vessels (black asterisks). Magnification: ×200; scale bar: 50 μm.

The immunohistochemical analysis of TNF-α expression in the CSBD of the rat calvaria is shown in Figure 6.

## 4. Discussion

The concept of utilizing LIPUS for bone repair has been established for decades. Numerous preclinical and clinical investigations have demonstrated accelerated bone healing which led to the implementation of LIPUS for fresh fractures as well as nonunions and delayed unions. All these findings, while plentiful, predominantly refer to long bone defects undergoing endochondral ossification. However, its efficacy in facilitating osteogenesis within bones that ossify intramembranously remains inconclusive.

Given the established efficacy of LIPUS as a non-invasive modality that enhances repair of long bone defects healing through endochondral ossification, we sought to investigate its therapeutic potential in intramembranous ossification. Being a representative model of intramembranous bone regeneration, we performed a CSBD, which represents the minimal osseous injury of the experimental animal that fails to undergo complete spontaneous repair. The outcomes were subsequently compared with those achieved using the current gold standard in reconstructive bone surgery—AB grafts.

Several studies have also evaluated the regenerative potential of LIPUS in rat calvarial defect models. Jung et al. reported approximately a 20% increase in bone formation after repeated LIPUS exposure in 4-mm defects [23]. Lavandier et al. demonstrated that a higher acoustic intensity (300 mW/cm^2^) significantly enhanced bone reconstruction in 3-mm defects, whereas 100 mW/cm^2^ showed no measurable effect [24]. Using 30 mW/cm^2^, Hasuike et al. observed nearly double the re-ossification rate compared with controls after four weeks [25]. Similarly, Imafuji et al. found that daily LIPUS stimulation increased ossification and bone volume, particularly when combined with collagen scaffolds and BMP-9 [26]. In all these studies, newly formed bone was quantified using computed tomography.

Nevertheless, robust experimental data directly comparing the efficacy of LIPUS with AB graft-mediated bone regeneration remain limited, especially in studies evaluating immunohistochemical markers such as TNF-α.

The findings of the present study, can be compared with studies examining the biological mechanisms of LIPUS in other regenerative models. One such study evaluated the influence of LIPUS on the chondrogenic differentiation of human umbilical cord–derived mesenchymal stem cells (hUC-MSCs) and cartilage repair in a rat articular cartilage defect model [27]. The authors reported that LIPUS enhanced the expression of cartilage-related markers and significantly promoted cartilage regeneration following MSC transplantation. Additionally, LIPUS stimulation reduced TNF-α gene expression, indicating a potential anti-inflammatory mechanism [27]. Despite these similarities, important differences exist between our study and the study of Chen et al. The referenced study focused on cartilage regeneration and MSC-mediated chondrogenic differentiation, whereas our research examined intramembranous bone healing in a rat calvarial critical-size defect model. Furthermore, while Chen et al. evaluated LIPUS primarily in combination with MSC transplantation, our study compared LIPUS with the current clinical gold standard for bone regeneration—AB grafting. Another distinction lies in the assessment of inflammatory signaling, as the previous study analyzed TNF-α expression mainly in vitro, whereas our study evaluated TNF-α expression directly within regenerating bone tissue in vivo using immunohistochemistry. Together, both studies support the concept that LIPUS may enhance tissue regeneration partly through modulation of inflammatory pathways.

In comparison with one of our previous studies, where we quantified newly formed bone by histomorphometry, each of the two bone quantification methods consistently demonstrated increased bone volume [28]. Both micro-CT from this study and bone histomorphometry from the previous study, demonstrated a progressive increase in newly formed bone over time, with a statistically significant difference at day 30 compared to days 7 and 15 in all groups (*p* < 0.001). The micro-CT analysis showed the highest bone volume in the AB group (26.83%), followed by the LIPUS group (23.74%), while the control group exhibited the lowest value (15.85%). A similar pattern was observed with histomorphometry, where the AB group reached 20.35%, the LIPUS group 19.12%, and the control group 5.11%. At day 30, both experimental groups demonstrated significantly greater bone volume compared to the control. The histomorphometric analysis revealed that LIPUS produced significantly higher BV/TV values at days 7 and 30 compared with the control group, whereas AB showed a significant increase only at day 30. In contrast, micro-CT detected statistically significant BV/TV differences among all three groups at each evaluated time point (days 7, 15, and 30) [28]. Furthermore, in our previous study, immunohistochemical analysis demonstrated that LIPUS significantly increased COX-2 expression during the early phases of healing (days 7 and 15), primarily in inflammatory cells, osteoprogenitors, and immature osteocytes. Increased Osterix expression was also observed, indicating enhanced osteogenic differentiation and bone formation [28]. In the present study, TNF-α expression exhibited a different temporal pattern, being initially highest in the control group and subsequently increasing in the LIPUS group at later healing stages. Given the complex role of TNF-α in bone biology, these findings should be interpreted with caution. When considered together with the previously reported COX-2 and Osterix findings, the present results suggest that LIPUS may influence multiple phases of bone healing, including early osteogenic responses and later remodeling-associated processes. Nevertheless, future investigations incorporating complementary inflammatory and osteogenic markers would help further clarify the biological significance of the observed TNF-α expression patterns.

Bone volume exhibited time-dependent and treatment-dependent variation. Across all groups, a progressive increase in bone formation was observed, with peak values recorded on postoperative day 30. Statistical analysis revealed significant differences between day 30 and the earlier evaluation points (days 7 and 15) in each group (*p* < 0.001) (Table 1; Figure 3). The quantitative micro-computed tomography (micro-CT) analysis demonstrated the highest percentage of newly formed bone in the AB group (26.83%), followed by the LIPUS group (23.74%), whereas the control group showed the lowest values (15.85%) (Table 1). By day 30, both experimental interventions resulted in significantly greater bone volume compared with spontaneous healing in the control group. Furthermore, statistically significant differences in BV/TV values were detected among all three groups at each analyzed time point (days 7, 15, and 30), as determined by micro-CT assessment (Table 1; Figure 3).

TNF-α plays a central role in the regulation of bone metabolism. Alongside RANKL, TNF-α promotes osteoclastogenesis and bone resorption; however, it does not independently induce osteoclast differentiation [29,30,31]. Numerous studies have reported an inhibitory effect of TNF-α on osteogenesis, primarily mediated through suppression of osteoblast differentiation. One mechanism by which TNF-α exerts this inhibition is the downregulation of key transcription factors such as Osx and Runx2, which are essential for osteoblast differentiation [32,33]. Nevertheless, the influence of TNF-α on the differentiation of mesenchymal stem cells (MSCs) into osteoblasts remains controversial in the literature. Some studies suggest that TNF-α inhibits MSC osteogenic differentiation via Notch signaling pathways [34,35], whereas others indicate that TNF-α can stimulate MSC osteogenesis through regulation of Runx2, Osx, and BMP-2 expression [36,37,38]. These seemingly paradoxical findings are largely attributed to variations in experimental conditions, particularly differences in TNF-α concentration and exposure duration, which can lead to opposing effects on cellular differentiation [39,40].

In our study, immunohistochemical analysis of TNF-α revealed that on day 7, expression was significantly higher in the control group compared to the AB and LIPUS groups. By day 15, TNF-α expression was higher in the LIPUS group, significantly differing from both the AB and control groups. On day 30, the LIPUS group still showed significantly higher TNF-α expression compared to the control group, but expression did not significantly differ from the AB group (Table 4). Given that TNF-α has been associated with regulation of bone remodeling processes through the RANK/RANKL/OPG pathway [39], these findings may indicate that LIPUS and AB influence inflammatory and repair-associated biological processes during different stages of intramembranous bone healing. Rather than indicating direct osteoclast activation, the observed TNF-α expression patterns likely reflect a broader regulation of the healing microenvironment, encompassing inflammatory and regenerative responses during bone formation. In the later phase of osteogenesis, TNF-α expression was observed in bone cells, especially preosteoblasts and osteoblasts at sites of bone apposition, as well as in multinucleated cells resembling osteoclasts (Figure 6).

From a translational perspective, the observed regenerative effects of LIPUS may be clinically relevant in conditions requiring intramembranous bone regeneration. Although direct extrapolation from animal models to human patients remains limited, the present findings support further investigation of LIPUS as a non-invasive adjunctive approach for enhancing bone repair in reconstructive and regenerative bone surgery.

Several limitations of this study should be considered when interpreting the findings. No formal a priori power calculation was performed, although statistically significant differences were detected for the primary study outcomes. In addition, blinding was not implemented during outcome assessment, which may have introduced observer bias. Furthermore, TNF-α was evaluated as the sole immunohistochemical marker in the present study, limiting the mechanistic interpretation of the observed findings. Finally, as this was an experimental animal study, caution is warranted when extrapolating the findings directly to clinical settings.

## 5. Conclusions

In conclusion, LIPUS and AB grafts stimulated osteogenesis, leading to a substantial increase in bone volume within the CSBD of the rat calvaria. Using 3D (micro-CT) analysis, the highest amounts of newly formed bone were observed in the AB group, followed by the LIPUS group, with the lowest values in the control group. Bone volume increased over time, reaching peak values on postoperative day 30, with statistically significant differences compared to earlier time points (days 7 and 15) in all groups. LIPUS enhanced tissue expression of the inflammatory marker TNF-α, while a similar temporal pattern was observed in the AB group. TNF-α expression increased in later stages, reflecting late-stage inflammatory and reparative activity during the healing process. These findings suggest that both LIPUS and AB modulate inflammatory and regenerative processes associated with intramembranous bone healing. Although AB grafting achieved the greatest bone volume, LIPUS demonstrated considerable regenerative potential and may represent a promising non-invasive therapeutic approach for enhancing intramembranous bone regeneration. These findings provide additional insight into LIPUS-mediated bone regeneration in intramembranous ossification, suggesting its potential utility as a non-invasive approach to support bone healing. However, further investigation is warranted to clarify the biological mechanisms underlying LIPUS-mediated bone repair and to optimize treatment protocols for maximal regenerative efficacy.

## Figures and Tables

**Table 1:** Micro-CT results of the BV/TV parameter (%). Data are presented as mean ± standard deviation (SD).

Day	LIPUS Group	Autologous Bone Group	Control Group	Overall *p*-Value *	Pairwise Differences **
7	14.81 ± 0.39	18.94 ± 1.85	11.92 ± 0.06	<0.001	cl, ca, al
15	20.78 ± 1.31	23.15 ± 0.36	13.13 ± 0.17	<0.001	cl, ca, al
30	23.74 ± 0.42	26.83 ± 0.28	15.85 ± 0.49	<0.001	cl, ca, al

* Analysis of variance. ** cl—statistically significant difference between the control and LIPUS groups. ca—statistically significant difference between the control and autologous bone groups. al—statistically significant difference between the autologous bone and LIPUS groups.

**Table 2:** Micro-CT results of the trabecular thickness (Tb.Th) in mm. Data are presented as mean ± standard deviation (SD).

Day	LIPUS Group	Autologous Bone Group	Control Group	Overall *p*-Value *	Pairwise Differences **
7	3.01 ± 0.09	3.95 ± 0.06	2.95 ± 0.04	<0.001	ca, al
15	4.71 ± 0.96	6.39 ± 0.26	3.09 ± 0.01	<0.001	cl, ca, al
30	4.86 ± 0.05	8.39 ± 0.22	3.51 ± 0.21	<0.001	cl, ca, al

* Analysis of variance. ** cl—statistically significant difference between the control and LIPUS groups. ca—statistically significant difference between the control and autologous bone groups. al—statistically significant difference between the autologous bone and LIPUS groups.

**Table 3:** Micro-CT results of the trabecular number (Tb.N). Data are presented as mean ± standard deviation (SD).

Day	LIPUS Group	Autologous Bone Group	Control Group	Overall *p*-Value *	Pairwise Differences **
7	0.017 ± 0.004	0.036 ± 0.004	0.008 ± 0.0008	<0.001	cl, ca, al
15	0.027 ± 0.006	0.048 ± 0.003	0.017 ± 0.003	<0.001	cl, ca, al
30	0.031 ± 0.002	0.054 ± 0.011	0.040 ± 0.006	0.010	al

* Analysis of variance. ** cl—statistically significant difference between the control and LIPUS groups. ca—statistically significant difference between the control and autologous bone groups. al—statistically significant difference between the autologous bone and LIPUS groups.

**Table 4:** Immunohistochemical intensity results. Data are presented as mean ± standard deviation (SD).

	Day	LIPUS Group	Autologous Bone Group	Control Group	Overall *p*-Value *	Pairwise Differences **
**TNF-α**	7	125.2 ± 0.8	133.8 ± 0.9	172.0 ± 1.1	<0.001	cl, ca, al
	15	137.7 ± 1.3	134.2 ± 1.8	126.6 ± 2.2	<0.001	cl, ca, al
	30	147.6 ± 1.9	146.3 ± 0.8	115.8 ± 0.9	<0.001	cl, ca

* Analysis of variance. ** cl—statistically significant difference between the control and LIPUS groups. ca—statistically significant difference between the control and autologous bone groups. al—statistically significant difference between the autologous bone and LIPUS groups.

## Data Availability

The data presented in this article are available on request from the corresponding author.
